# Supplementary material for: Actin-microtubule interplay coordinates spindle assembly in human oocytes
Source: Nat Commun. 2019 Oct 11;10:4651. doi: 10.1038/s41467-019-12674-9 (PMC6789129; doi:10.1038/s41467-019-12674-9)
Supplement: Supplementary file 1 — Supplementary Information [file 41467_2019_12674_MOESM1_ESM.pdf]

Supplementary Information

# Actin-microtubule interplay coordinates spindle assembly in human oocytes

Roeles and Tsiavaliaris

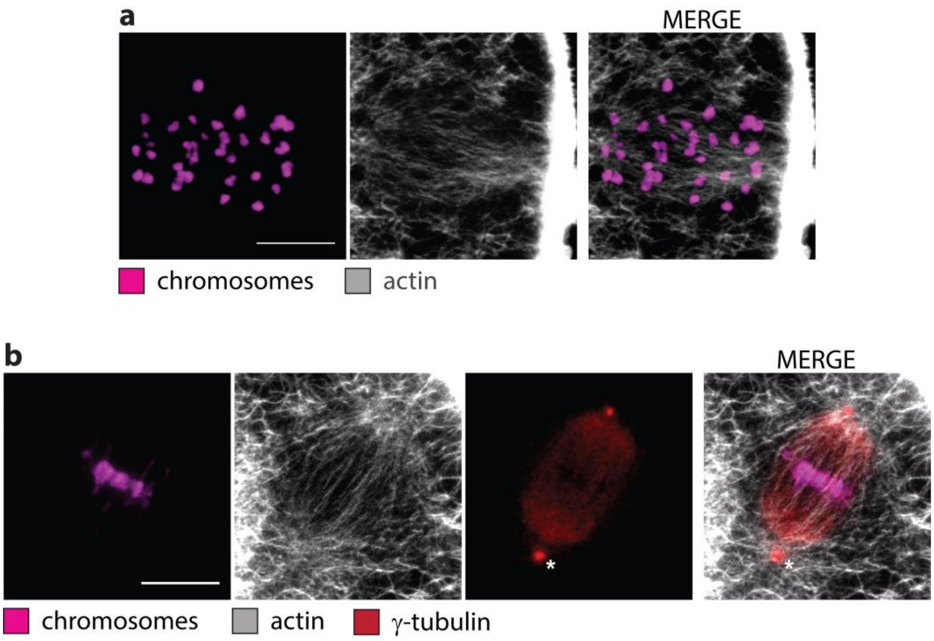


**Supplementary Fig. 1** Actin pervades the spindle of oocytes with abnormal chromosome number and associates with microtubules surrounding sperm DNA. **a**, Representative z-projection of a diploid metaphase II oocyte stained for chromosomes (Hoechst) and actin (phalloidin); z = 25 sections. **b**, Immunofluorescence staining of γ-tubulin together with chromosomes (Hoechst) and actin (phalloidin) at a spindle structure surrounding the sperm DNA in an ICSI treated oocyte. Asterisk marks sperm centrosome; z = 13 sections. Scale bars, 10 µm.

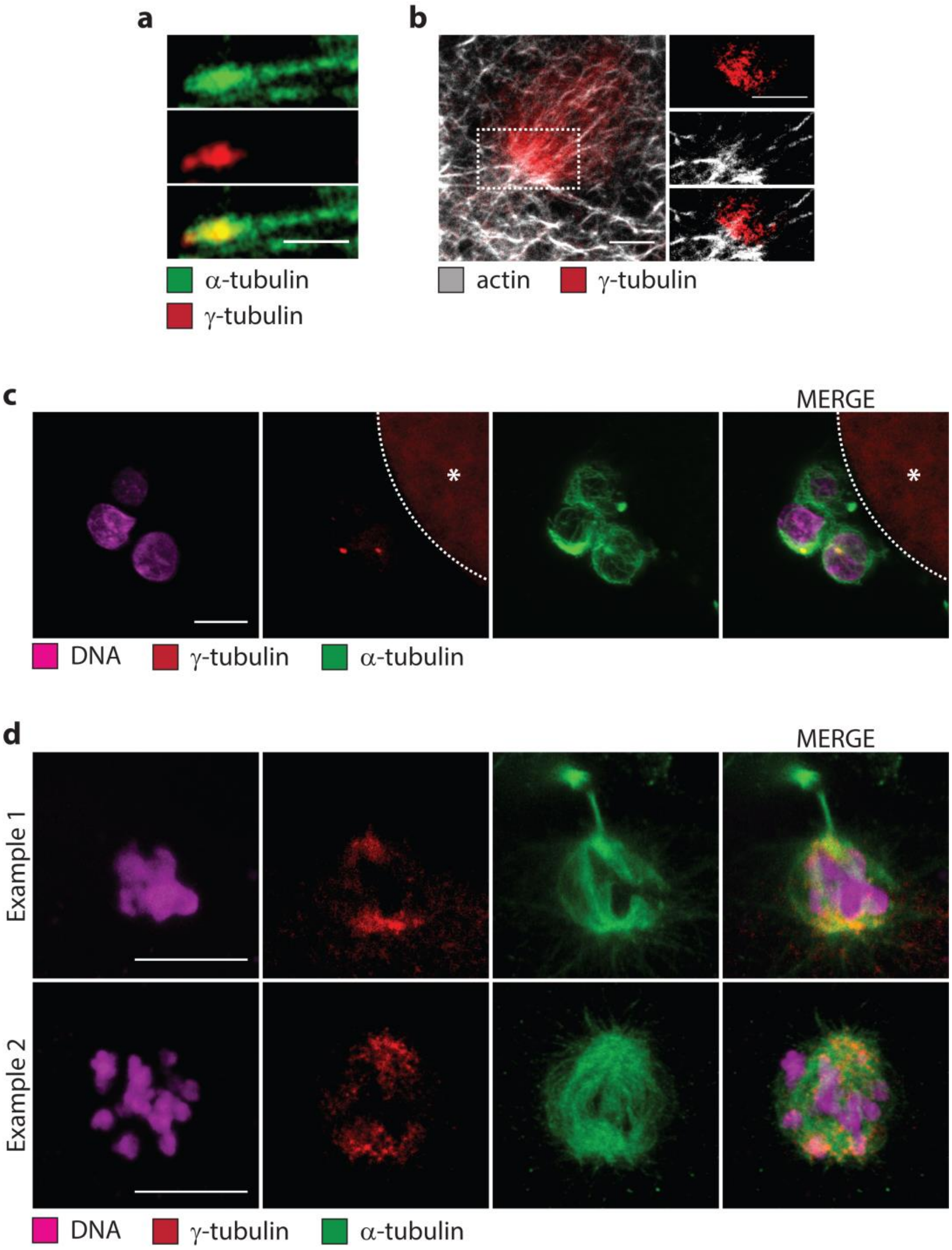


**Supplementary Fig. 2** Characterization of γ-tubulin distribution in human oocytes. **a**, Immunofluorescence image showing microtubules (α-tubulin) and γ-tubulin. Scale bar, 2 µm. **b**, Pole region of a metaphase II oocyte showing γ-tubulin and actin (phalloidin); z = 7 sections. Scale bar, 5 µm. **c**, Distribution of γ-tubulin in oocyte cytoplasm and cumulus cells. Immunofluorescence staining shows DNA (Hoechst), microtubules (α-tubulin) and γ-tubulin. Asterisk marks oocyte; z = 15 sections. Scale bar, 10 µm. **d**, Immunofluorescence projections showing chromosomes (Hoechst), microtubules (α-tubulin) and γ-tubulin in oocytes at early meiosis II; z = 11 sections. Scale bars, 10 µm.

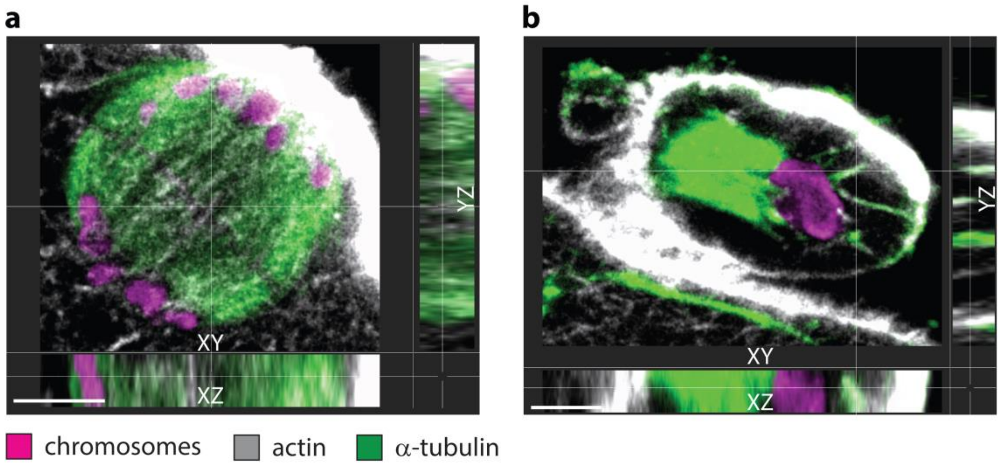


**Supplementary Fig. 3** Actin and microtubules adopt distinct and overlapping distribution patterns. **a** and **b**, Orthogonal sections showing chromosomes (Hoechst), actin (phalloidin) and microtubules (α-tubulin) inside the centre volume of anaphase I (**a**) and telophase I (**b**) spindles. Depth (*z*) = 3 µm. Scale bars, 10 µm.

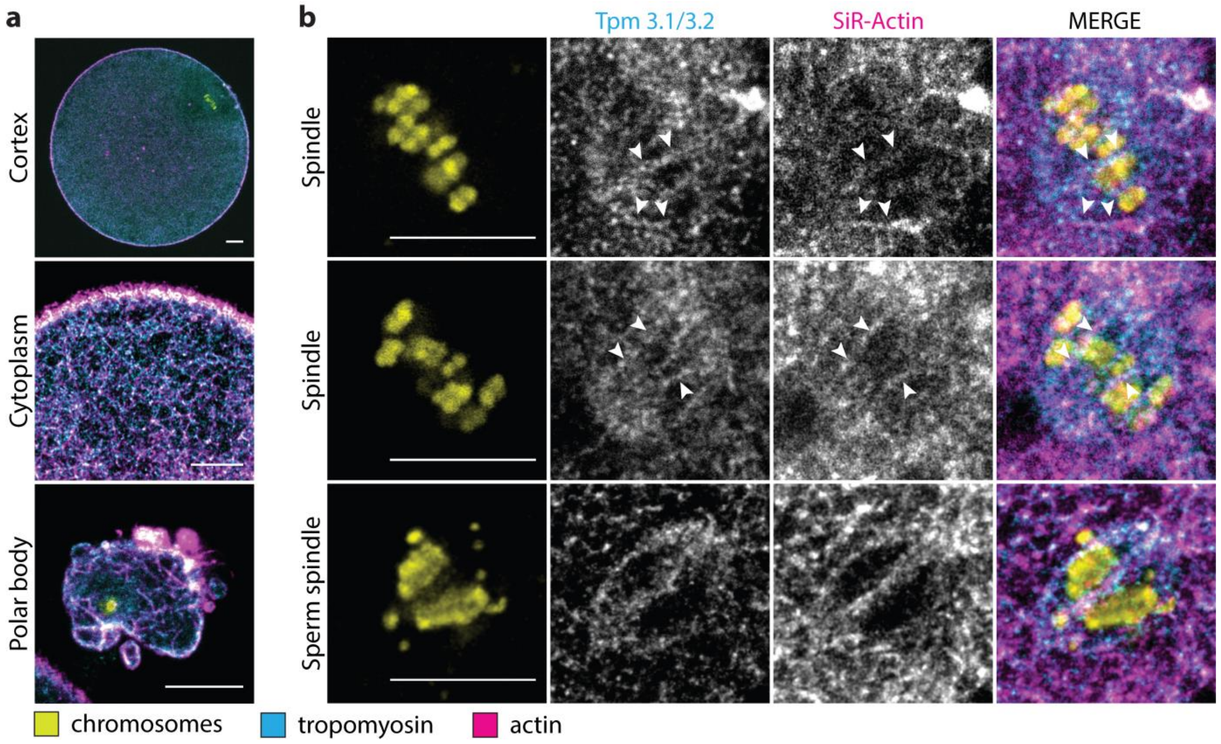


**Supplementary Fig. 4** Spindle actin is associated with tropomyosins in human oocytes. **a**, Representative images showing tropomyosin Tpm3.1/3.2 and actin (SiR-actin) together with chromosomes (Hoechst) at the cortex, cytoplasm and polar body of metaphase II oocytes; z = 1, 2 and 1 sections. **b**, Close-up view of Tpm3.1/3.2 and actin (SiR-actin) at spindles surrounding maternal and paternal (sperm) chromatin in ICSI treated oocytes; z = 3, 4 and 3 sections. Arrowheads mark signal overlap at the spindle. Scale bars, 10 µm.

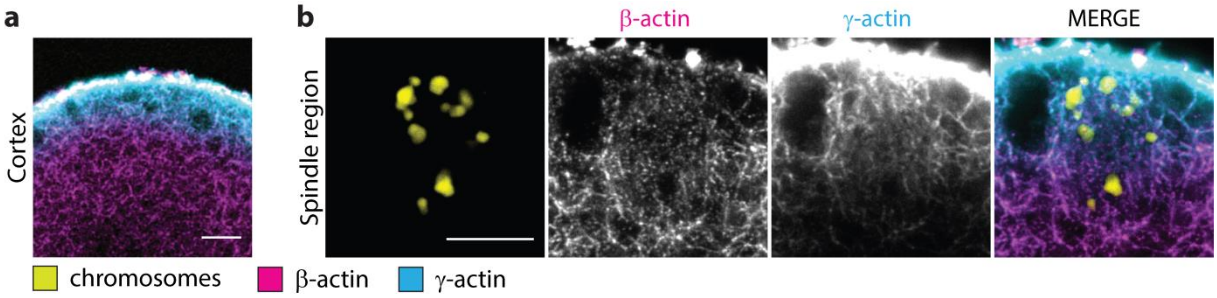


**Supplementary Fig. 5** Different specific actin isoforms meet in the vicinity of the meiotic spindle. **a** and **b**, Total view and spindle close-up of metaphase II oocytes stained for chromosomes (Hoechst), β-actin and γ-actin; z = 1 and 9 sections. Scale bars, 10 µm.

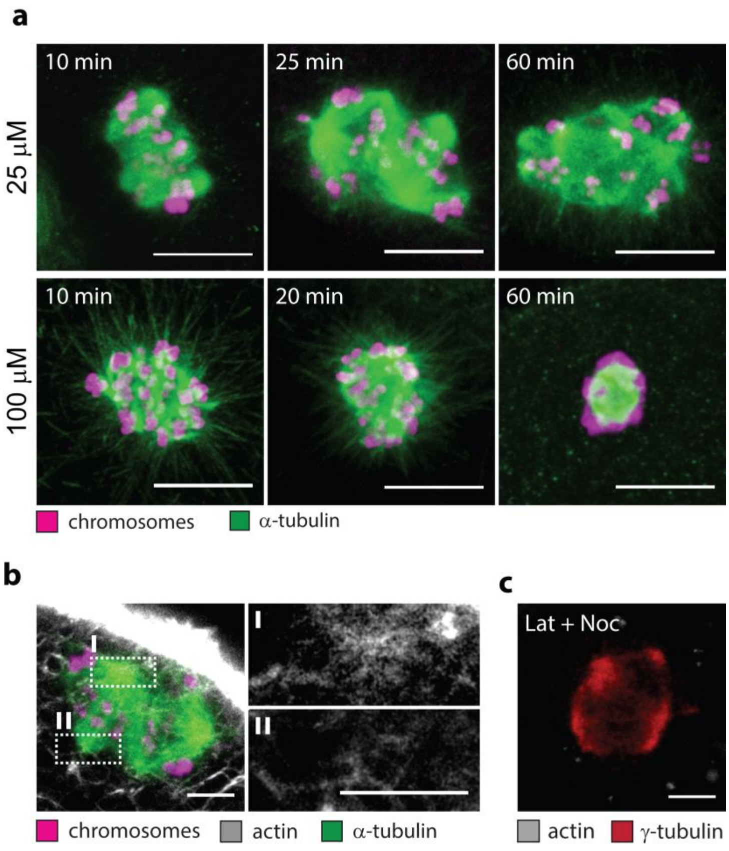


**Supplementary Fig. 6** Microtubule decomposition by nocodazole affects spindle actin organization. **a**, Immunofluorescence projections exemplifying chromosome and microtubule arrangement in nocodazole-treated oocytes. z = 11 sections. Scale bars, 10 μm. **b**, Close-up view of actin accumulations (phalloidin) at different multipoles of a nocodazole-treated oocyte stained for chromosomes (Hoechst), actin (phalloidin) and α-tubulin; z = 4 sections. Scale bars, 5 μm. **c**, Representative image of an oocyte treated with latrunculin-B and subsequently incubated in both latrunculin-B and nocodazole showing actin (phalloidin) and γ-tubulin; z = 5 sections. Scale bar, 5 μm.

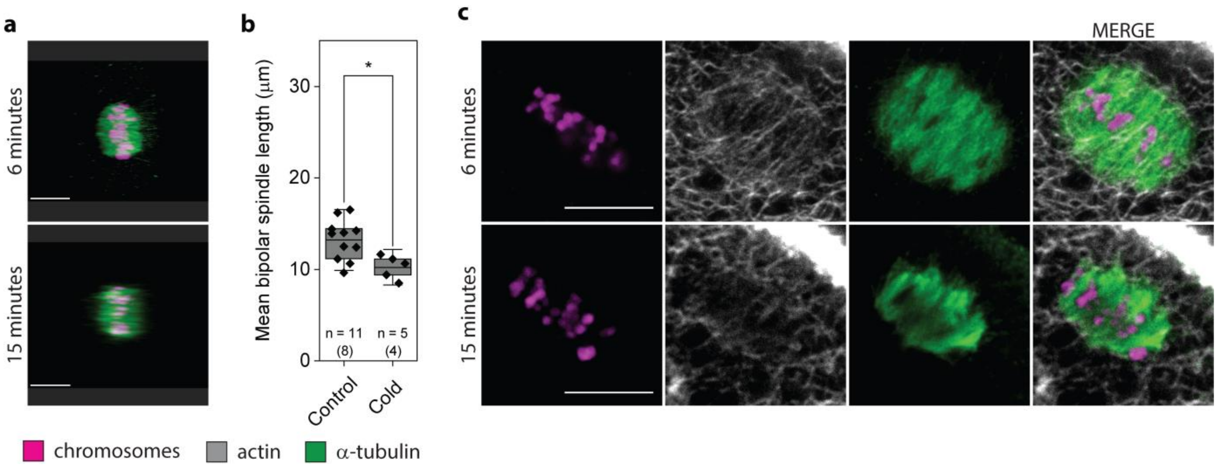


**Supplementary Fig. 7** Microtubule decomposition by cold affects spindle actin organization. **a**, 3D snapshots of chromosomes (Hoechst) and microtubules (α-tubulin) exemplifying short spindles with broad poles in cold-treated oocytes. Scale bars, 10 µm. **b**, Mean length of bipolar spindles in untreated MII oocytes and oocytes exposed to cold for 6 to 15 minutes. Control measurements are equal to those shown in Fig. 3b. **c**, Example z-projections of cold-treated oocytes after 6 and 10 minutes showing chromosomes (Hoechst), actin (phalloidin) and α-tubulin; z = 3 and 4 sections. Scale bars, 10 µm. n = total number of oocytes. The number of donors is specified within parentheses. Centre lines are mean values; boxes illustrate 25th and 75th percentiles. Error bars represent standard deviation. Significance was tested using the two-tailed two sample t-test. Asterisk denotes p-value: * < 0.05. Source data are provided as a Source Data file.

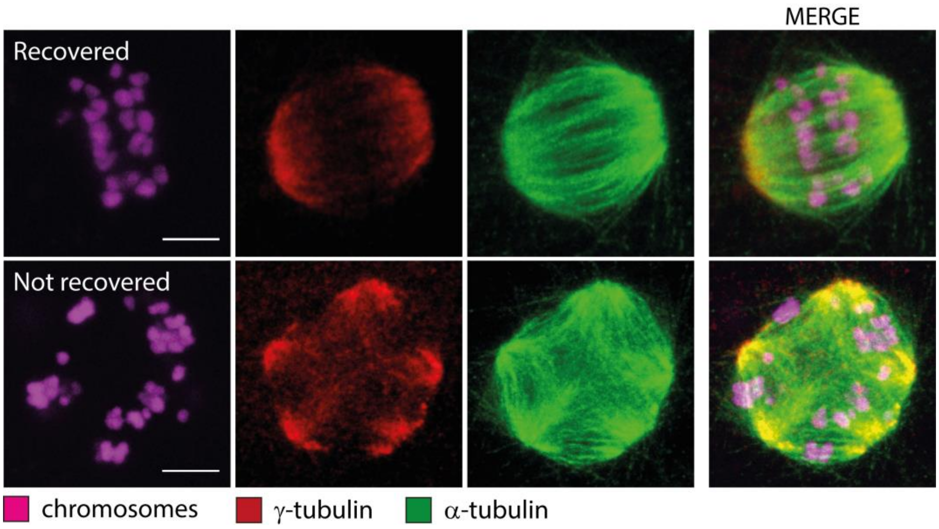


**Supplementary Fig. 8** γ-tubulin shows bipolar distribution in recovered oocytes. Representative z-projections of recovered and not recovered MII oocytes after nocodazole treatment and 60 to 90 minutes regeneration time showing chromosomes (Hoechst), α-tubulin and γ-tubulin; z = 4 sections. Scale bars, 5 µm.
