# Supplementary material for: Actin-microtubule interplay coordinates spindle assembly in human oocytes
Source: Nat Commun. 2019 Oct 11;10:4651. doi: 10.1038/s41467-019-12674-9 (PMC6789129; doi:10.1038/s41467-019-12674-9)
Supplement: Supplementary file 3 — Description of Additional Supplementary Files [file 41467_2019_12674_MOESM3_ESM.pdf]

Description of Additional Supplementary Files

File name: Supplementary Movie 1

Description: 3D reconstruction of actin filaments within the spindle | Three-dimensional animation showing original actin signal and filament reconstruction within the spindle of a human metaphase II oocyte stained for chromosomes (Hoechst, magenta) and actin (phalloidin, grey). The z stack contained z = 19 confocal sections, taken every 0.5 µm. Volume reconstruction was performed using Imaris software. Scale bar, 5 µm.

File name: Supplementary Movie 2

Description: Microtubules attach to the spindle poles via multiple γ-tubulin foci | Three-dimensional animation showing microtubules (α-tubulin, green) and γ-tubulin (red) in a human metaphase II oocyte. The z stack contained z = 22 confocal sections, taken every 0.5 µm. Volume reconstruction was performed using Imaris software. Scale bar, 10 µm.

File name: Supplementary Movie 3

Description: Actin filaments within the anaphase I spindle | Animated z-stack of a human oocyte at anaphase I stained for chromosomes (Hoechst, magenta) and actin (phalloidin, grey) generated with ImageJ software. Confocal sections were taken every 0.5 µm. Scale bar, 5 µm.

File name: Supplementary Movie 4

Description: Actin filaments within the telophase I spindle | Animated z-stack of a human oocyte at telophase I stained for chromosomes (Hoechst, magenta) and actin (phalloidin, grey) generated with ImageJ software. Confocal sections were taken every 0.5 µm. Scale bar, 5 µm.

File name: Supplementary Movie 5

Description: Actin filaments within the metaphase II spindle | Animated z-stack of a human oocyte at metaphase II stained for chromosomes (Hoechst, magenta) and actin (phalloidin, grey) generated with ImageJ software. Confocal sections were taken every 0.5 µm. Scale bar, 5 µm.

File name: Supplementary Movie 6

Description: Monastrol leads to loss of bipolar microtubule alignment and causes γ-tubulin defocusing | Three-dimensional animation showing chromosomes (Hoechst, magenta), microtubules (α-tubulin, green) and γ-tubulin (red) in a human metaphase II oocyte treated with monastrol. The z stack contained z = 21 confocal sections, taken every 0.5 µm. Volume reconstruction was performed using Imaris software. Scale bar, 10 µm.

File name: Supplementary Movie 7

Description: Nocodazole induces spindle multipolarity and splitting of γ-tubulin clusters | Three-dimensional animation showing chromosomes (Hoechst, magenta), microtubules (α-tubulin, green) and γ-tubulin (red) in a human metaphase II oocyte treated with nocodazole. The z stack contained z = 23 confocal sections, taken every 0.5 µm. Volume reconstruction was performed using Imaris software. Scale bar, 10 µm.

File name: Supplementary Movie 8

Description: Subsequent treatment with nocodazole and taxol leads to large multipolar spindles | Three-dimensional animation showing chromosomes (Hoechst, magenta), microtubules (α-tubulin, green) and γ-tubulin (red) in a human metaphase II oocyte subsequently treated with nocodazole and taxol. The z stack contained z = 46 confocal sections, taken every 0.5 µm. Volume reconstruction was performed using Imaris software. Scale bar, 15 µm.

File name: Supplementary Movie 9

Description: Subsequent treatment with monastrol and taxol leads to large multipolar spindles | Three-dimensional animation showing chromosomes (Hoechst, magenta), microtubules (α-tubulin, green) and γ-tubulin (red) in a human metaphase II oocyte subsequently treated with monastrol and taxol. The z stack contained z = 43 confocal sections, taken every 0.5 µm. Volume reconstruction was performed using Imaris software. Scale bar, 15 µm.

File name: Supplementary Movie 10

Description: Actin at the multipoles is preserved after subsequent treatment with monastrol and taxol | Three-dimensional animation showing chromosomes (Hoechst, magenta), microtubules (α-tubulin, green), γ-tubulin (red) and actin (phalloidin, grey) at one multipole of a human metaphase II oocyte subsequently treated with monastrol and taxol. The z stack contained z = 12 confocal sections, taken every 0.5 µm. Volume reconstruction was performed using Imaris software. Scale bar, 10 µm.
