# Supplementary material for: Actin-microtubule interplay coordinates spindle assembly in human oocytes
Source: Nat Commun. 2019 Oct 11;10:4651. doi: 10.1038/s41467-019-12674-9 (PMC6789129; doi:10.1038/s41467-019-12674-9)
Supplement: Supplementary file 14 — Reporting Summary [file 41467_2019_12674_MOESM14_ESM.pdf]

nature research

Corresponding author(s): Georgios Tsiavaliaris

Last updated by author(s): 27.8.2019

Reporting Summary

Nature Research wishes to improve the reproducibility of the work that we publish. This form provides structure for consistency and transparency in reporting. For further information on Nature Research policies, see Authors & Referees and the Editorial Policy Checklist .

Statistics

For all statistical analyses, confirm that the following items are present in the figure legend, table legend, main text, or Methods section.

n/a	Confirmed	
☐	☒	The exact sample size (*n*) for each experimental group/condition, given as a discrete number and unit of measurement
☐	☒	A statement on whether measurements were taken from distinct samples or whether the same sample was measured repeatedly
☐	☒	The statistical test(s) used AND whether they are one- or two-sided<br>*Only common tests should be described solely by name; describe more complex techniques in the Methods section.*
☒	☐	A description of all covariates tested
☒	☐	A description of any assumptions or corrections, such as tests of normality and adjustment for multiple comparisons
☐	☒	A full description of the statistical parameters including central tendency (e.g. means) or other basic estimates (e.g. regression coefficient) AND variation (e.g. standard deviation) or associated estimates of uncertainty (e.g. confidence intervals)
☐	☒	For null hypothesis testing, the test statistic (e.g. *F*, *t*, *r*) with confidence intervals, effect sizes, degrees of freedom and *P* value noted<br>*Give P values as exact values whenever suitable.*
☒	☐	For Bayesian analysis, information on the choice of priors and Markov chain Monte Carlo settings
☒	☐	For hierarchical and complex designs, identification of the appropriate level for tests and full reporting of outcomes
☒	☐	Estimates of effect sizes (e.g. Cohen's *d*, Pearson's *r*), indicating how they were calculated

Our web collection on statistics for biologists contains articles on many of the points above.

Software and code

Policy information about availability of computer code

Data collection	Leica Application Suite X
Data analysis	ImageJ Version 2.0.0<br>Imaris (Bitplane) Version 8.2.1<br>Origin Software 2018, 64 bit (Originlab)<br>Microsoft Excel 2010<br>MatLab R2017a (Mathworks)

For manuscripts utilizing custom algorithms or software that are central to the research but not yet described in published literature, software must be made available to editors/reviewers. We strongly encourage code deposition in a community repository (e.g. GitHub). See the Nature Research guidelines for submitting code & software for further information.

Data

Policy information about availability of data

All manuscripts must include a data availability statement. This statement should provide the following information, where applicable:
- Accession codes, unique identifiers, or web links for publicly available datasets
- A list of figures that have associated raw data
- A description of any restrictions on data availability

The authors declare that the data supporting the findings of this study are available within the paper and its supplementary information files. The source data underlying Figures 3b, 3d-e, 3g, 4d, 5a, 5c and Supplementary Figure 7d are provided as a Source Data file.

Field-specific reporting

Please select the one below that is the best fit for your research. If you are not sure, read the appropriate sections before making your selection.

- [x] Life sciences
- [ ] Behavioural & social sciences
- [ ] Ecological, evolutionary & environmental sciences

For a reference copy of the document with all sections, see nature.com/documents/nr-reporting-summary-flat.pdf

Life sciences study design

All studies must disclose on these points even when the disclosure is negative.

Sample size	Sample size was chosen on the basis of available oocytes donated by women who gave their informed consent (> 500 oocytes from 184 women)
Data exclusions	Data were only excluded for failed experiments or because of unsuitable quality of the oocyte for deeper experimental investigations.
Replication	Replicate experiments were successful and reliably reproduced.
Randomization	Randomization was not performed because not applicable.
Blinding	Investigators were blinded to all donor-related parameters except of age. Investigators were totally blind towards the data sampling.

Reporting for specific materials, systems and methods

We require information from authors about some types of materials, experimental systems and methods used in many studies. Here, indicate whether each material, system or method listed is relevant to your study. If you are not sure if a list item applies to your research, read the appropriate section before selecting a response.

Materials & experimental systems

n/a	Involved in the study	
☐	☒	Antibodies
☒	☐	Eukaryotic cell lines
☒	☐	Palaeontology
☒	☐	Animals and other organisms
☒	☐	Human research participants
☒	☐	Clinical data

Methods

n/a	Involved in the study	
☒	☐	ChIP-seq
☒	☐	Flow cytometry
☒	☐	MRI-based neuroimaging

Antibodies

Antibodies used	- anti-alpha-tubulin monoclonal antibody [Molecular Probes A11126]<br>- goat anti-mouse Alexa Fluor 488 [Molecular Probes A11029]<br>- goat anti-mouse Alexa Fluor 647 [Molecular Probes A21235]<br>- goat anti-mouse IgG1 Alexa Fluor 488 antibody [Jackson Immunoresearch 115-545-205]<br>- anti-gamma tubulin antibody [Santa Cruz Biotechnology sc17788]<br>- goat-anti mouse IgG2b Cy5 antibody [Jackson Immunoresearch 115-175-207]<br>- anti-gamma9d antibody [Merck Millipore AB5447]<br>- donkey anti-sheep Cy3 antibody [Merck Millipore AP184C]<br>- anti-beta-actin antibodies [Nordic MUbio MUB0110P]<br>- anti-gamma-actin antibody [Nordic MUbio MUB0111P]
Validation	All antibodies in this study have been validated using model cell lines and within different oocyte samples. For detailed description of the conditions used please refer to methods section.
